# Genomic diversity and phylogeography of norovirus in China

**DOI:** 10.1186/s12920-017-0287-9

**Published:** 2017-10-03

**Authors:** Niu Qiao, He Ren, Lei Liu

**Affiliations:** 1 Key Laboratory of Medical Molecular Virology of MoE & MoH and Institutes of Biomedical Sciences, Shanghai Medical College, Fudan University, 138 Yi Xue Yuan Rd, Shanghai, 200032 People’s Republic of China; 2 Shanghai University of Medicine & Health Sciences, Shanghai, 201318 People’s Republic of China

**Keywords:** Phylogeography, Norovirus, Bayesian phylogenetics, Demographic dynamics, China

## Abstract

**Background:**

Little is known about the phylogeography of norovirus (NoV) in China. In norovirus, a clear understanding for the characteristics of tree topology, migration patterns and its demographic dynamics in viral circulation are needed to identify its prevalence trends, which can help us better prepare for its epidemics as well as develop useful control strategies. The aim of this study was to explore the genetic diversity, temporal distribution, demographic dynamics and migration patterns of NoV that circulated in China.

**Results:**

Our analysis showed that two major genogroups, GI and GII, were identified in China, in which GII.3, GII.4 and GII.17 accounted for the majority with a total proportion around 70%. Our demography inference suggested that during the long-term migration process, NoV evolved into multiple lineages and then experienced a selective sweep, which reduced its genetic diversity. The phylogeography results suggested that the norovirus may have originated form the South China (Hong Kong and Guangdong), followed by multicenter direction outbreaks across the country.

**Conclusions:**

From these analyses, we indicate that domestic poultry trade and frequent communications of people from different regions have all contributed to the spread of the NoV in China. Together with recent advances in phylogeographic inference, our researches also provide powerful illustrations of how coalescent-based methods can extract adequate information in molecular epidemiology.

**Electronic supplementary material:**

The online version of this article (doi:10.1186/s12920-017-0287-9) contains supplementary material, which is available to authorized users.

## Background

Norovirus (NoV) is the commonest causative agent of gastroenteritis worldwide, which has high morbidity and mortality, especially in children, the elderly and patients with immunosuppression and low immune function [1–3]. NoV can cause recurrent bouts of vomiting, diarrhea, abdominal pain and low-grade fever that typically last 24–48 h, while one third showed latent infection [4]. NoV shedding can be prolonged for several weeks and decreased gradually with the processing of time [5]. Norovirus is extremely contagious, and transmission occurs by three general routes: food-borne outbreaks, water-borne outbreaks and person-to-person transmission [6, 7]. Food-borne and water-borne transmissions are the primary causes of NoV outbreaks, which typically occur by ingestion of contaminated food products and faeces-contaminated water [8]. Person-to-person transmission is without a doubt the dominant transmission route for NoV, which might occur directly through the fecal-oral route and aerosol transmission [6, 8]. Given the above mentioned characteristics of NoV and there is no long lasting immunity to NoV, mass infection and outbreaks can occur in a variety of enclosed or semi enclosed institutional settings, such as hospitals, patrol fleets, nursing homes, schools, entertainment places and kindergartens [7, 9]. In China, the first isolation of norovirus was in Hong Kong in the mid 1970s [10]. Subsequently, the virus rapidly spread and became the leading cause of gastroenteritis across many different regions of China [10, 11].

Previous work on the NoV sequences used statistical and bioinformatics analysis to construct phylogenetic relationships and calculate the evolutionary rates of NoV but did not take into account the geographic dispersion between different regions within the country [11–13]. Phylogeography is the study of the geographic distribution patterns of lineages as well as the formation principles and processes of lineages [14]. BEAST Phylogeography analysis of influenza virus had been widely and thoroughly studied around the world [15–18]. Genetically, NoV is classified into six genogroups (GI-GVI) according to the genetic characteristics. Two major genogroups, GI and GII, mainly cause human acute gastroenteritis [19]. According to literature reports, NoV has similar epidemic pattern and evolutionary rate as influenza virus [20]. As with influenza virus, we can also explore the evolutionary history of NoV spread, including different genogrous of NoV.

The NoV was widely distributed in different regions of China [11] and recombination occasionally occurred resulting in new mutation variants, posting a serious disease burden [21]. In the past decade, the genetic and antigenic evolution of the NoV is well documented [22]. However, the geographic diffusion of NoV is poorly understood in China. A clear understanding of the tree topology, geographic spread and migration patterns of NoV are needed to identify its prevalence trends, which can help us better prepare for its epidemics as well as develop useful control strategies. Here, we explore the genetic diversity, temporal distribution, demographic and phylogeographic history of NoV that circulated in China using sequences obtained from GenBank through a Bayesian phylogeography coalescent approach [15].

## Methods

### Sequences and sequence analysis

All available NoV gene sequences in China isolated from human host were downloaded from the NCBI GenBank Database [23] by searching the corresponding taxonomy ID of noroviruses (Taxonomy ID: 142,786) from human host with a keyword “China” (accessed on May 24, 2016). The time and location of each sequence were retrieved from GenBank Database or the publication that corresponding to the sequence, and were used to estimate evolutionary rate and spatio-temporal evolutionary relationships, respectively. Sequences containing sampling time and locations were obtained for the study and were genotyped using an automated online NoV genotyping tool based on regions A, B, C, D, and E offered by the Netherlands National Institute for Public Health and the Environment (RIVM) [24].

To reduce the number of sequences and maintain enough phylogeographic information at the same time, we sampled sequences from each NoV genogroup and took one strain per year and per location. Besides, we also remove the recombination strain during our sampling process. Thus, our final GI dataset comprised 102 sequences and the GII dataset comprised 374 sequences (Additional file 1 in the supplemental material). All sequences analyzed in this study were then aligned using the multiple sequence alignment program MUSCLE [25] implemented by MEGA software version 6.06 [26]. Besides, manual editing was carried out to truncate the sequences at both 5′ and 3′ ends.

### Bayesian discrete phylodynamic and phylogeography analyses

To understand the spatial temporal dynamics of NoV, phylogeographic reconstruction was implemented in BEAST software v1.8.2 [27], which utilized a continuous time Bayesian Markov Chain Monte Carlo sampling over discrete sampling locations and applied a Bayesian stochastic search variable selection (BSSVS) model [15]. The rate of nucleotide substitution, the demographic history, the spatial location reconstruction and viral migration event were jointly estimated according to the following steps.

Firstly, to determine the best-fit model of nucleotide substitutions for both datasets, jModelTest v2.1.10 program [28] was implemented according to Akaike Information Criterion (AIC). A General Time Reversible (GTR) [29] nucleotide substitution model with gamma-distributed rate variation [30] among sites (GTR + G) was selected for both GI and GII NoV datasets. Then, the corresponding dates and parameters were assigned using the BEAUTi application [27], part of the BEAST package. Three different molecular clock models were used, either assuming a constant rate of evolution across the tree (strict molecular clock model), or modeling a molecular rate that varies among lineages (uncorrelated log-normal and uncorrelated exponential derivation models) [31]. A flexible coalescent demographic tree prior named Bayesian Skyline was selected to estimate the NoV evolutionary rate in the MCMC simulations [30, 32].

In all cases, the MCMC analysis was performed three times each for 100 million generations, sampling every 10000th generation for each data set (GI and GII). The convergence of continuous parameters was assessed by calculating the Effective Sample Size (ESS) (greater than 200) using the TRACER v1.6 program (http://tree.bio.ed.ac.uk/software/tracer/) after excluding the initial 10% of the run. Statistical uncertainty in parameter values across the sampled trees was given by the 95% highest probability density (HPD) values.

### Statistical phylogeography

To evaluate the posterior location uncertainty for the phylogeographic model, Kullback-Leibler (KL) divergence was calculated using “flexmix” package [33, 34] R version 3.2.0 (http://www.R-project.org/.), which is a measure of difference between the root state posterior and prior probabilities for each MCC tree [15]. The larger the KL value, the stronger root state statistical power the model produces. To calculate the KL divergence, we assume the prior distribution is a uniform discrete distribution.

To explore the association significance between a particular trait and its distribution on a phylogeny, Association Index (AI) and Parsimony Score (PS) test [15] were implemented using the program Bayesian Tip-Significance testing (BaTS) [35], which is a Bayesian MCMC approach and corrects for phylogenetic uncertainty. Statistical significance was defined as *p* < 0.05. The AI and PS test takes into account the shape of the phylogeny by measuring the imbalance of internal phylogeny nodes. The lower the AI and PS are, the stronger correlation of phylogeny-trait association is.

To provide statistical support for significant transmission routes between discrete locations, Bayes factor (BF) test [15] was conducted for both GI and GII sequences using SPREAD v1.0.6 software [36]. The higher the BF value is, the more likely that a migration may exist between two locations. Thus, we consider BF > 3 as well supported diffusion route in the migration graph.

### Visualizing phylogeographic diffusion

To summarize the spatial and temporal posterior distribution of ancestral location states, the Maximum Clade Credibility (MCC) trees for GI and GII NoVs were summarized with 10% chain removed as burn-in using TreeAnnotator v1.8.2, which is also a part of the BEAST package. The annotated MCC trees were constructed and visualized with FigTree v1.8.0 program (http://beast.bio.ed.ac.uk/figtree). To elucidate the viral effective population size through time, Bayesian Skyline Plot (BSP) [32] with a Stepwise constant variant for population growth was generated by Tracer v1.6 (http://tree.bio.ed.ac.uk/software/tracer/). To visualize the spatiotemporal transmission routes for both GI and GII NoVs in China, the MCC trees were converted into a keyhole markup language (KML) file using SPREAD v1.0.6 software [36], which is suitable for visualization with Google Earth (http://earth.google.com). Example KML files for both GI and GII genes are included as supplementary files (Additional files 2 and 3).

In summary, the overall framework of the analysis can be summarized as follows. Both GI and GII sequences were aligned via MUSCLE [25], nucleotide substitution models were tested via jModeltest [28], and Bayesian phylogeographic trees were created via BEAST [27]. Then, the maximum clade credibility (MCC) tree was summarized via TreeAnnotator [27]., Bayes Factor (BF) test was implemented using SPREAD [36], and Association Index (AI) and Parsimony Score (PS) test were implemented using the program Bayesian Tip-Significance testing (BaTS) [35]. Lastly, the MCC trees were visualized with FigTree (http://beast.bio.ed.ac.uk/figtree), Bayesian Skyline Plot (BSP) [32] with a Stepwise constant variant for population growth was generated by Tracer (http://tree.bio.ed.ac.uk/software/tracer/) and the keyhole markup language (KML) file was visualized via SPREAD [36].

## Results

### Genetic diversity and temporal distribution of NoV

A total of 3794 NoV sequences from China were download from GenBank Database (accessed on May 24, 2016). Of these 3794 sequences, 3648 were from human host, and 3134 of those containing sampling time and location were selected for the study.

**Fig. 1:**
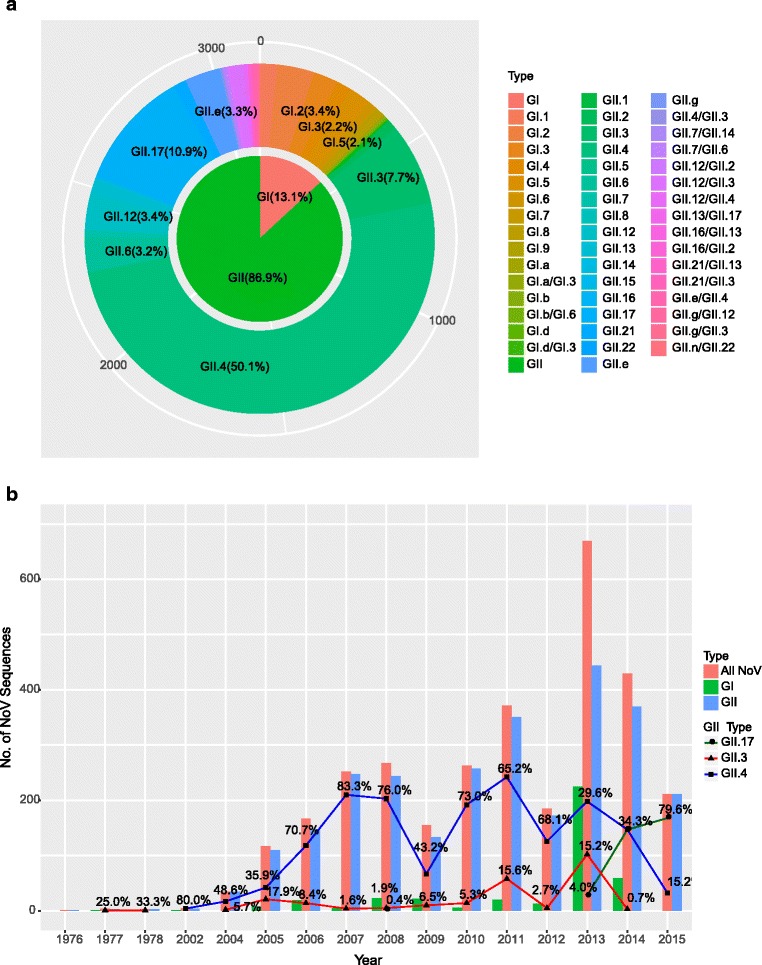
Distribution of NoV sequences reported to the GenBank between 1976 and 2015 (*n* = 3134). **a** Nested pie chart of the NoV genotype distribution proportions. **b** Distribution of NoV sequences. All NoV sequences were grouped by year and divided into “All NoV”, “GI”, “GII”, “GII.3”, “GII.4” and “GII.17”. The solid line with symbol represents the percentages of “GII.3”, “GII.4” and “GII.17” for the years shown

Overall, there were two NoV genogroups in China, including GI (411; 13.1%) and GII (2723; 86.9%) (Fig. 1a). Recombination analysis showed that a large majority (3016; 96.2%) of those NoV sequences were non recombinant strains and only a small portion (118; 3.8%) belonged to the recombinant strains (Additional file 4A in the supplemental material). Genotyping of these sequences revealed the presence of 12 GI genotypes and 18 GII genotypes, which mainly includes GI.1 (51; 1.6%), GI.2 (108; 3.4%), GI.3 (70; 2.2%), GI.4 (44; 1.4%), GI.5 (65; 2.1%) and the GII genotpye includes GII.3 (240; 7.7%), GII.4 (1571; 50.1%), GII.6 (99; 3.2%), GII.e (104; 3.3%), GII.12 (107; 3.4%), GII.13 (39; 1.2%), GII.17 (343; 10.9%), GII.21 (37; 1.2%), respectively (Fig. 1a).

The number of NoV sequences varied from 1 to 669 each year. And a large proportion more than 8% per year includes 2008 (267; 8.5%), 2010 (263; 8.4%), 2011 (371; 11.8%), 2013 (669; 21.5%) and 2014 (429; 13.7%). Among the GII genogroup, the GII.4 genotype had the highest prevalence in this study, followed by the genotype GII.17 and GII.3. The number of GII.4 sequences ranged from 4 to 242 by year with two remarkable increase in 2005 to 2007 (42 to 210) and 2009 to 2011 (67 to 242). The number of GII.3 NoV was relatively small with two minor peaks detected in 2011 (58) and 2013 (102). Notably, GII.17 has been growing rapidly since 2013, increased from initial 4.0% to 79.6% in 2015 (Fig. 1b).

In total, 11 GII.4 variants were identified in different provinces of China during the period of 2002–2015. The most prevalent variants were Den Haag_2006b (918), in 2006–2014, New Orleans_2009 (87) in 2009–2012, and Sydney_2012 (434) in 2012–2015; the three minor variants were Farmington_Hills_2002 (2), Lanzou_2002 (4), Asia_2003 (41), Kaiso_2003 (31), Hunter_2004 (31), Yerseke_2006a (2), Osaka_2007 (2) and Apeldoorn_2007 (1) (Additional file 4B in the supplemental material).

### Geographical distribution of NoV sequences

The noroviruses were widely but unequally geographically distributed in most regions of China (Additional file 5 in the supplemental material). The number of sequences from Beijing was the highest (765; 24.4%) among all the regions, followed by Shandong (562; 17.9%), Hong Kong (373; 11.9%), Guangdong (364; 11.6%) and Zhejiang (229; 7.3%). Obviously, the NoV sequences were collected from the coastal regions, such as Jilin, Liaoning, Beijing, Tianjin, Shandong, Jiangsu, Shanghai, Zhejiang, Fujian, Guangdong, Hong Kong, Guangxi and Hainan, which accounted for only one-fifth of the whole China. Besides, no NoV sequences were obtained from areas in West-China, such as Xinjiang, Inner Mongolia, Qinghai, Tibet and so on (Additional file 5 in the supplemental material).

### Phylogeography reconstruction of NoV in China

All of the nucleotide substitution rates and times of most recent common ancestor (tMRCA) are summarized in Table 1. The estimated nucleotide substitution rates by the uncorrelated lognormal clock model for GI was 1.69 × 10^−3^ substitutions/site/year (95% highest posterior density (HPD): 7.86 × 10^−4^ ~ 2.73 × 10^−3^), and for GII was 4.56 × 10^−3^ substitutions/site/year (95% HPD: 3.39 × 10^−3^ ~ 5.78 × 10^−3^). Molecular clock analyses showed that the estimated time to the most recent common ancestor (tMRCA) for GI and GII were around July 1738 (95% HPD: 1529 to NoV 1901) and Aug 1842 (95% HPD: July 1710 to Oct 1941), respectively (Table 1).

**Table 1:** Nucleotide substitution rates and divergence times for the VP1 genes of GI (*n* = 102, 1623 nt) and GII (*n* = 374, 1623 nt) NoVs in China^a^

Genotype	Molecular clock	Nucleotide substitution rate (10–3 substitutions/site/year)	TMRCA by:	
			No. of year	Date (range)
GI	Strict	1.22 (0.67, 1.80)	287.0 (159.1, 454.1)	1727.0 (1559.9, 1854.9)
GI	UCLN	1.69 (0.79, 2.73)	275.4 (112.1, 485.0)	1738.6 (1529.0, 1901.9)
GI	UCED	1.68 (0.88, 2.62)	276.9 (100.2, 515.1)	1737.1 (1498.9, 1913.8)
GII	Strict	2.69 (2.12, 3.31)	191.3 (133.4, 253.3)	1823.7 (1761.7, 1881.6)
GII	UCLN	4.56 (3.39, 5.78)	172.3 (74.0, 304.4)	1842.7 (1710.6, 1941.0)
GII	UCED	4.47 (3.47, 5.50)	151.4 (65.5, 280.1)	1863.6 (1734.9, 1949.5)

^a^The nucleotide substitution rate is the mean rate for the three individual determinations. *UCLN* uncorrelated log-normal clock, *UCED* uncorrelated exponential deviation clock, *TMRCA* time to most recent common ancestor. Values in parentheses are 95% HPDs

**Fig. 2:**
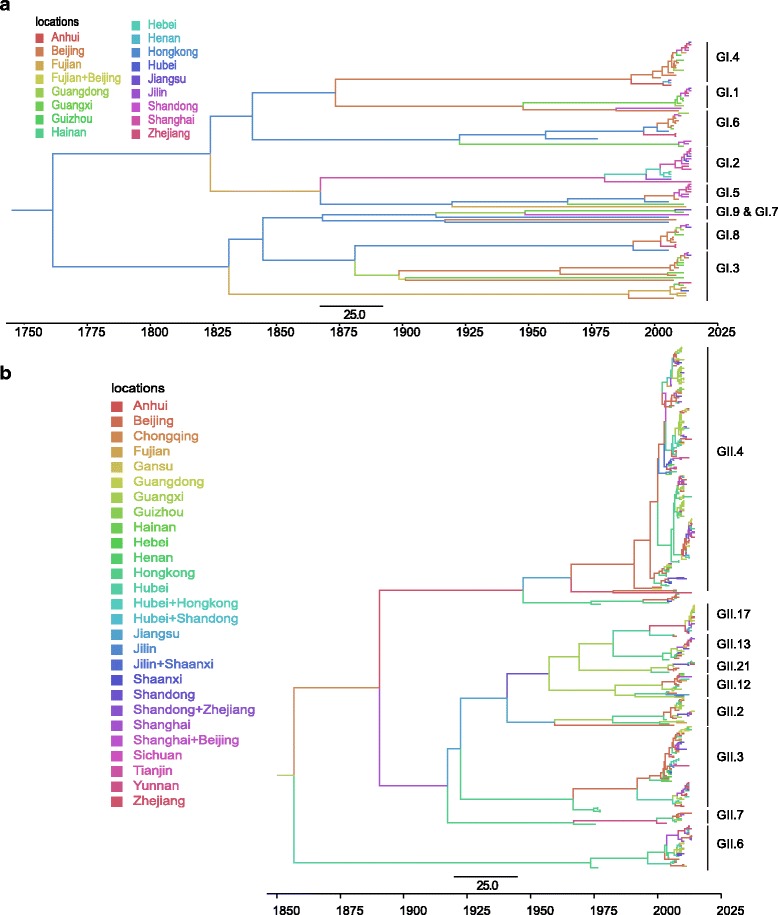
Maximum clade credibility (MCC) phylogenies of NoV GI (**a**) and GII (**b**) in China. Branches are colored according to the most probable location state of their descendent nodes. The scale bar in the bottom indicates the years before the most recent sampling time (2015)

From the MCC tree (Fig. 2a), we can inferred that the phylogenetic relationship of GI.4, GI.1, GI.6, GI.2 and GI.5 were much closer since they were located in one of the major branch. Likewise, GI.5, GI.9, GI.7, GI.8 and GI.3 were located in another major branch. The genealogical tree of GII NoV (Fig. 2b) was mainly divided into three major branches. Among them, GII.6 was located in the earliest branch, the most prevalent genotype GII.4 was located in one of the main braches, and the third branch was composed of GII.17, GII.13, GII.21, GII.12, GII.2, GII.3 and GII.7. Besides, our time estimation also indicated that wide migration and vast genetic diversification of the NoV occurred from approximately 2000 to 2015, during which the virus might migrated from Hong Kong and Guangdong to many other inland provinces (Fig. 2).

**Fig. 3:**
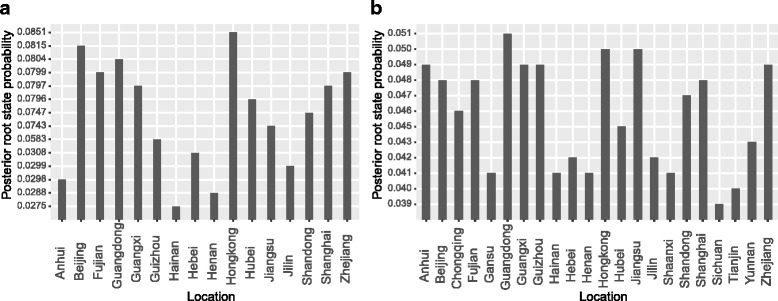
Posterior root state probability calculated from GI (**a**) and GII (**b**) MCC phylogenies. The histogram shows the posterior probability distributions of root location state of GI and GII

The spatial reconstruction of GI (Fig. 3a) indicated that Hong Kong as the origin with the highest root state posterior probability 0.0851. However, as for GII NoV, most probable root state agreed with Guangdong as origin instead of Hong Kong (Fig. 3b). It is worth discussing that the posterior probability for both GI and GII is less than 0.1, suggesting the uncertainty of the NoV transition.

In addition, we calculated the Kullback-Leibler (KL) divergence to estimate the divergence between the root state prior and posterior probabilities for both GI and GII NoVs. We used a fixed prior of 1/K for each tree, where K is the number of unique states. The GII tree yields lower KL divergence than that of the GI tree (Table 2), indicating a much smaller deviation of the posterior distribution of the root location from the prior. Besides, the observed Association Index (AI) and Parsimony Scores (PS) for both GI and GII NoVs are highly significant (Table 2), signifying that there is a strong phylogeny-locality correlation during the process of virus evolution.

**Table 2:** Summary statistics of phylogeography metrics for the GI (*n* = 102, 1623 nt) and GII (*n* = 374, 1623 nt) NoVs in China^a^

Genotype	Molecular clock	KL	Association index			Parsimony score		
			Observed	Expected	*P* value	Observed	Expected	*P* value
GI	Strict	0.070	7.87 (6.96, 8.78)	8.95 (8.32, 9.46)	0.004	65.92 (64.00, 68.00)	70.87 (67.74, 73.84)	0.004
GI	UCLN	0.077	8.04 (7.11, 8.95)	9.19 (8.58, 9.73)	0.006	65.63 (64.00, 68.00)	70.85 (67.76, 73.67)	0.006
GI	UCED	0.096	8.05 (7.13, 8.98)	9.20 (8.58, 9.71)	0.002	65.66 (64.00, 68.00)	70.77 (67.75, 73.43)	0.006
GII	Strict	0.008	28.67 (26.75, 30.53)	37.73 (36.62, 38.75)	0.000	221.20 (215.00, 227.00)	273.05 (266.96, 278.76)	0.000
GII	UCLN	0.004	28.53 (26.57, 30.46)	37.55 (36.49, 38.59)	0.000	218.40 (212.00, 224.00)	272.91 (267.52, 278.29)	0.000
GII	UCED	0.009	28.54 (26.58, 30.57)	37.56 (36.39, 38.59)	0.000	218.67 (213.00, 225.00)	272.95 (267.31, 278.30)	0.000

^a^UCLN, uncorrelated log-normal clock. *UCED* uncorrelated exponential deviation clock. Values in parentheses are 95% HPDs. Both GI and GII had an AI and PS *p*-value <0.05

### Population dynamics during NoV geographic diffusion process

**Fig. 4:**
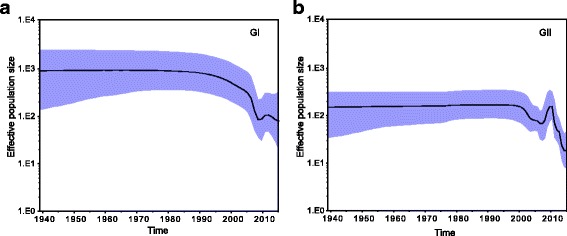
Bayesian skyline plots of NoV in China estimated from GI (**a**) and GII (**b**) VP1 sequences. The plots illustrate the relative effective population size (genetic diversity) of GI and GII NoVs through time. The solid black line represents the mean posterior value and the blue area corresponds to the 95% highest probability density (HPD) intervals

To explore the changes in genetic diversity over time of NoV in China, we inferred its demographic history through a Bayesian Skyline Plots (BSP) coalescent model. Figure 4 shows a marked difference in the evolution dynamics of these two genogroups, in which temporal changes of effective population size were plotted. The effect population size for both genogroups remained constant from 1940 continuously through earlier 2000. GII experienced a slowly decrease in earlier 2000 after which the population increase rapidly with a maximum peak around 2010. Following the peak, genetic diversity declined gradually. Additionally, GI do not show an increase peak of activity after 2005 but rather a decrease circulation of genetic variants (Fig. 4).

### Spatiotemporal dynamics of NoV geographic dispersal

**Fig. 5:**
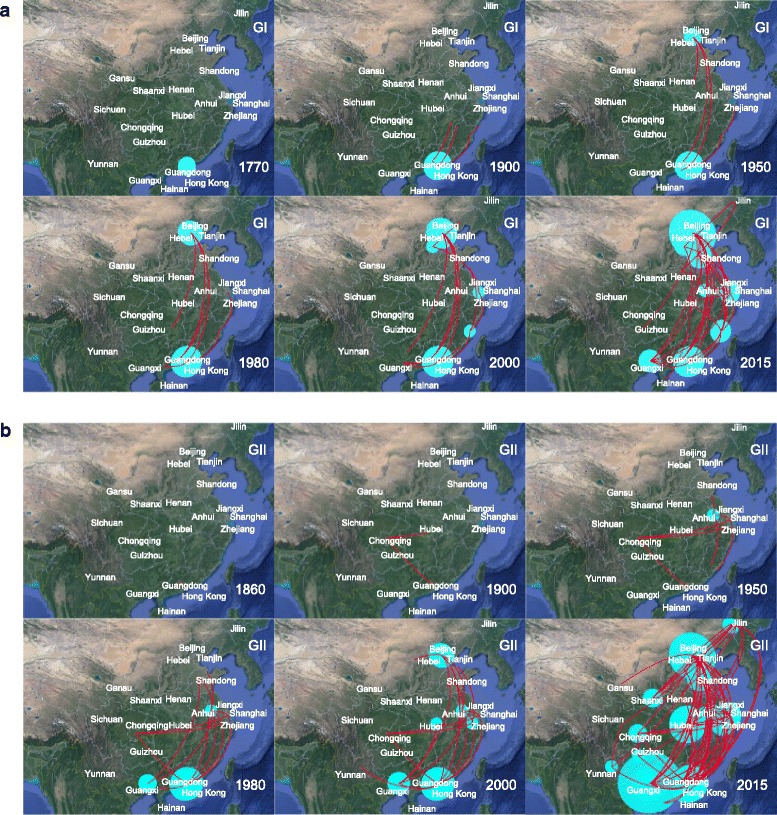
Spatiotemporal dispersal of NoV among different localities of China reconstructed using discrete phylogeographic analysis of GI (**a**) and GII (**b**). Snapshots of the dispersal pattern of GI and GII were provided for 1700, 1900, 1950, 1980, 2000, 2015 and 1860, 1900, 1950, 1980, 2000, 2015, respectively. The red lines represented MCC phylogeny branches projected on the surface, whereas the uncertainty on the locations of NoV was represented by cyan polygons. The 95% HPD regions were obtained by imputing locations on each branch across the posterior distribution using bivariate kernel density estimates for the respective sample. The maps are based on satellite pictures made available in Google Earth (http://earth.google.com)

To understand the domestic spread progress of NoV in China along with nature time scales, we visualized the annotated phylogeographic MCC trees with estimated divergence times and spatial information for both GI and GII on Google Earth. Figure 5 showed the spatiotemporal dynamics of NoV dispersal process in China. The red lines connecting different geographic locations represent branches in the MCC tree on which virus transmission occur and circles area represent the number of branches maintaining a particular location at that time point.

When combined with the results for both GI and GII genogroups, the NoV might originated in Guangdong and Hong Kong before 1860 (Fig. 5). During the next few years, the virus continued to accumulate and showed signs of transmission. By 1950, the earliest dispersal events occurred in which the virus spread from Hong Kong to the northeast along with the coast regions (Fujian, Zhejiang, Shanghai and Jiangxi). Then, the virus continued to spread into the north of China (Hebei, Beijing and Tianjin) by 1980. The diffusion process intensified by 2000 which propagated the virus to the west and southwest neighboring provinces of Guangxi, Yunan, Guizhou and Chongqing as well as to the central China (Hubei). Although some controlling effort had been made, the virus continued circulating in China. Finally, the norovirus disseminated most areas of China in a short period of time with a major transmission wave and even spread into Gansu and Jilin by 2015. The map indicated that the early overall migration patterns of the norovirus are roughly from South to North, followed by multicenter direction outbreaks across the country.

**Fig. 6:**
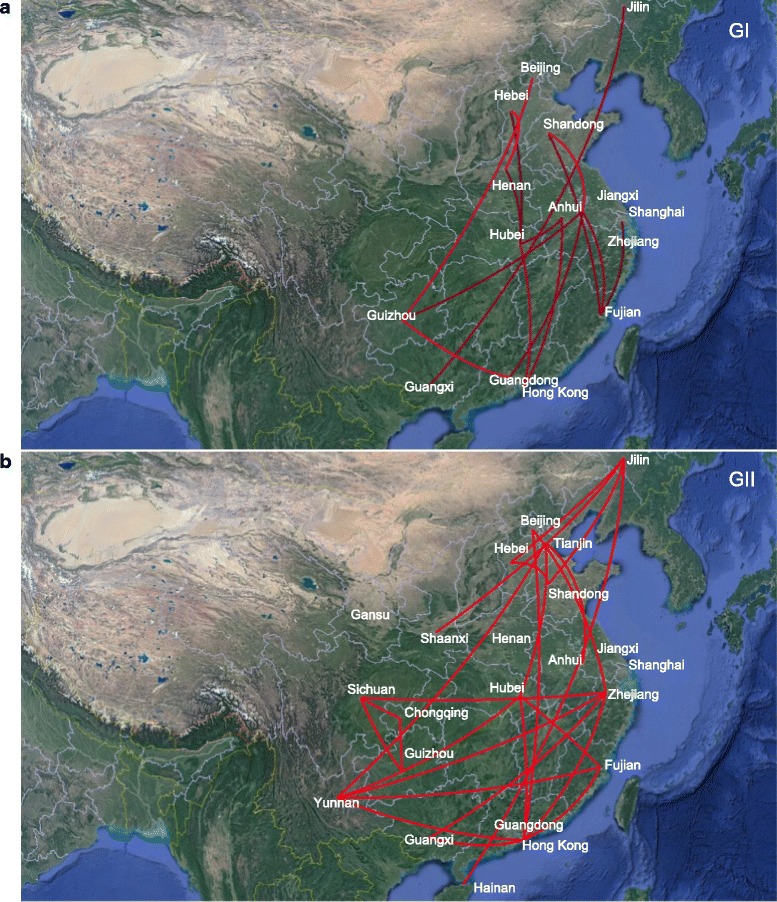
Bayesian factor (BF) test for significant non-zero dispersion routes in NoV GI (**a**) and GII (**b**). Only routes supported by a BF greater than 3 are plotted. The line color represent the relative strength by which the routes are supported: dark red lines and bright red lines suggest weak and strong support respectively. The maps are based on satellite pictures made available in Google Earth (http://earth.google.com)

To identify statistically significant transmission routes between discrete locations, we conducted Bayes factor (BF) test for the most significant non-zero rates. We used a BF cutoff of three to define the significance (Additional file 1 in the supplemental material). Fifteen significant GI routes were identified, and the migration graph is markedly parsimonious with a distinctive South to North propagation trend. As for GII, 24 well supported routes were found, and a majority of the migration links was related to Hong Kong, Zhejiang, Yunan, Hubei and Jilin (Fig. 6b). The transmission pattern for GII was much more complicated when compared to GI (Fig. 6a). In the south of China, Hong Kong was closely related to six locations (Beijing, Fujian, Guangxi, Zhejiang, Yunan and Hebei). In the east of China, Zhejiang was related to five provinces (Beijing, Guangxi, Hong Kong, Sichuan and Yunan). In the southwest of China, Yunan was also related to five locations (Tianjin, Zhejiang, Fujian, Hong Kong and Hubei). In the northeast of China, Jilin was linked to four regions (Shandong, Shaanxi, Hainan and Hebei). In the central of China, Hebei was also linked to four regions (Yunna, Tianjin, Fujian and Hong Kong). The difference between the two genogroups is most likely due to larger uncertainty in the root state estimation for GI and GII (Fig. 3).

## Discussion

To investigate the geographic spread pattern of NoV in China, a Bayesian phylogeography inference framework was applied for both GI and GII NoVs sequences sets. We reconstructed the time-scale phylogeographic Maximum Clade Credibility (MCC) trees, in which each branch is assigned different colors to represent different probable locations and the calibrating time-scale is shown above the horizontal axis. We also inferred the ancestral locations of NoV through the root state posterior probability shown in Fig. 3. Most of the NoV sequences obtained from distinct geographical regions in China appeared to be closely related based on the phylogenetic analysis instead of grouping into distinct lineages according to geographical locations. Our above analysis suggests that southern China (Guangdong and Hong Kong) is the original source of norovirus. While migrating through the entire country, major lineages of GII NoV were generated from 2005 to 2010, as supported in BSP analysis in which the effective population size increased rapidly during this time period. Our inference is consistent with the previous reports during which the epidemic variants of NoV arose globally [20].

Our spatial and temporal dynamics for the geographic spread of the NoV in China revealed more details about its migration patterns. Our analysis for the GI and GII showed that the early overall migration patterns of the norovirus are roughly from South to North along with the coastal regions, then different simultaneous migration events occurred in various directions rather than a unidirectional pattern, and most of these migrations are short-range. Besides, approximately 90.27% (2829/3134) of NoV sequences were collected from the coastal regions. One plausible speculation for the results mentioned above is linked to NoV-contaminated seafood, for example domestic trade of contaminated oysters [8]. On the other hand, coastal areas of China have obvious geographical advantages with developed economy, comparatively, and most sentinel hospitals were located in these provinces, which may lead to more studies about NoV. Besides, high population density and frequent communications of people from different regions (for instance, the Spring Festival and Migrant workers) across the country may also attribute to the multicenter spread of NoV as well as increase the opportunity of NoV cross infection [8, 37]. Moreover, the absence of NoV variants in some regions may result from the lack of surveillance studies.

The demographic plots can help elucidate a demographic history by showing increases and decreases in genetic diversity within a defined time frame. The MCMC results showed that the relaxed clock model was a significantly better fit model than the strict clock model for both of the datasets, thus the subsequent analyses were mainly generated by uncorrelated lognormal model. Our analysis confirmed the existence of several distinct demographic periods during NoV expansion. Before 2005, the overall genetic diversity among strains was lower for the GII than for GI NoVs, after which the GII population increase rapidly with a maximum peak around 2010. This is consistently shown in the phylogenetic MCC trees, in which the GI genotypes are less distant from one another compared with the GII genotypes. Our Bayesian coalescent evolutionary analysis estimated that the NoV VP1 capsid gene evolved at a mean rate of 1.69 × 10^−3^ and 4.56 × 10^−3^ nucleotide substitutions/site/year by uncorrelated lognormal molecular clock models for the GI and GII NoVs, respectively. According to literature reports, most RNA viruses evolve at a rate of approximately 10^−3^ nucleotide substitutions/site/year [38]. Additionally, RNA viruses have the ability to experience genetic changes to persist in human populations [39]. Therefore, our results suggest that the overall lower prevalence of GI compared to GII NoVs between 2005 and 2015 cannot be attributed to differences in the rate of nucleotide evolution in the VP1 region, since the rates of evolution between the two genogroups were similar for both strict and relaxed molecular clock models.

Many questions in evolutionary biology require a bio-geographical perspective on the population under investigation. As mentioned above, most of the NoV sequences were collected from the coastal regions of China. The evolutionary rate, evolutionary time scale and demographic history of NoV in China only require the sampling time information. However, the spatial dynamics of norovirus in China rely on both the sampling time and location of each sequence, which may cause potential analyzing bias. In order to minimize the sampling bias, all available NoV sequences in China were downloaded from GenBank Database. Besides, we sampled sequences from each NoV genogroup and took one strain per year and per location to maintain enough phylogeographic information. We hope to have demonstrated the Bayesian framework can contribute significantly to evolutionary molecular epidemiology.

There are limitations to the result of our analysis. Firstly, the Bayesian inference framework requires the researcher first to carry out a Bayesian MCMC analysis of the data, which can be time and memory consuming. Secondly, the origin predicted (Guandong or Hong Kong) has probability of less than 0.1, though higher than other cities, it’s hard to draw a definite conclusion that Guandong or Hong Kong is the origin for NoV. Deliberate and powerful evidence is needed in our future research to gain new insights into the viral migration and evolutionary dynamics in China.

## Conclusions

We examined for the first time the spatial dynamics of NoV genogroups, GI and GII, to explore the phylogeography of norovirus in China. We analyzed the genetic diversity, temporal distribution, demographic history and the spatial diffusion pattern of norovirus that circulated in China by using a Bayesian coalescent framework. Our analysis showed that two major genogroups, GI and GII, were identified in China, in which GII.3, GII.4 and GII.17 accounted for the majority with a total proportion around 70%. Our demographic history reveals that during the long-term migration process, NoV evolved into multiple lineages and the overall genetic diversity among strains is lower for the GII than for GI NoV before 2005 after that GII had a maximum peak around 2010. Our phylogeography result suggests that norovirus might originate from southern China (Guangdong and Hong Kong) and subsequently spread from the south to north along with the coastal areas, followed by multi direction and multicenter propagation. Our results provide powerful illustrations of how coalescent-based methods can extract adequate information in molecular epidemiology. In the future, we will continue to gain new insights into the viral migration and evolutionary dynamics in China.

## Additional files

Additional file 1: GenBank accession numbers and Bayes factor test results of GI and GII NoVs sequences used in this study. (PDF 395 kb)

Additional file 2: KML file for NoV migration among locations of China over time as inferred from GI. Can be opened with Google Earth for visualization or any text editor or editing. (PDF 720 kb)

Additional file 3: KML file for NoV migration among locations of China over time as inferred from GII. Can be opened with Google Earth for visualization or any text editor or editing. (XLS 696 kb)

Additional file 4: Distribution of NoV in China. (A) Pie chart of the recombinant (Re) and non-recombination (Non-Re) strains distribution ratio of NoV in China during 1976–2015 (*n* = 3134). (B) Temporal distribution of NoV GII.4 variants in China between 1976 and 2015 (*n* = 1553). (KML 4347 kb)

Additional file 5: Geographical distribution of NoV in China between 1976 and 2015 (*n* = 3134). (KML 14978 kb)

## Abbreviations

AI: Association index
AIC: Akaike information criterion
BaTS: Bayesian tip-significance testing
BF: Bayes factor
BSP: Bayesian skyline plot
BSSVS: Bayesian stochastic search variable selection
ESS: Effective sample size
GTR: General time reversible
HPD: Highest probability density
KL: Kullback-leibler
KML: Keyhole markup language
MCC: Maximum clade credibility
PS: Parsimony score
RIVM: Netherlands national institute for public health and the environment
