# Supplementary figures and images for: Genomic diversity and phylogeography of norovirus in China
Source: BMC Med Genomics. 2017 Oct 3;10(Suppl 3):51. doi: 10.1186/s12920-017-0287-9 (PMC5629611; doi:10.1186/s12920-017-0287-9)

A

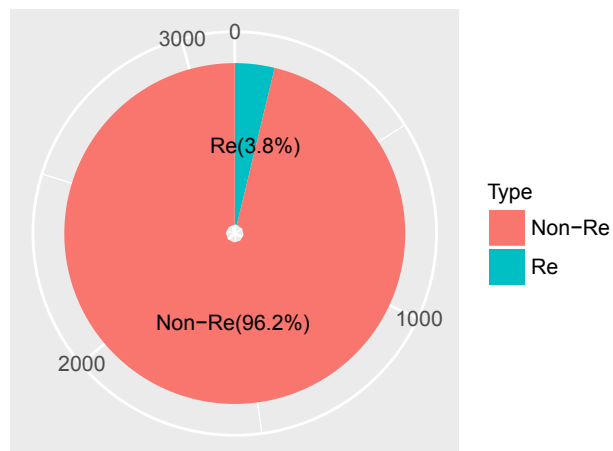

B

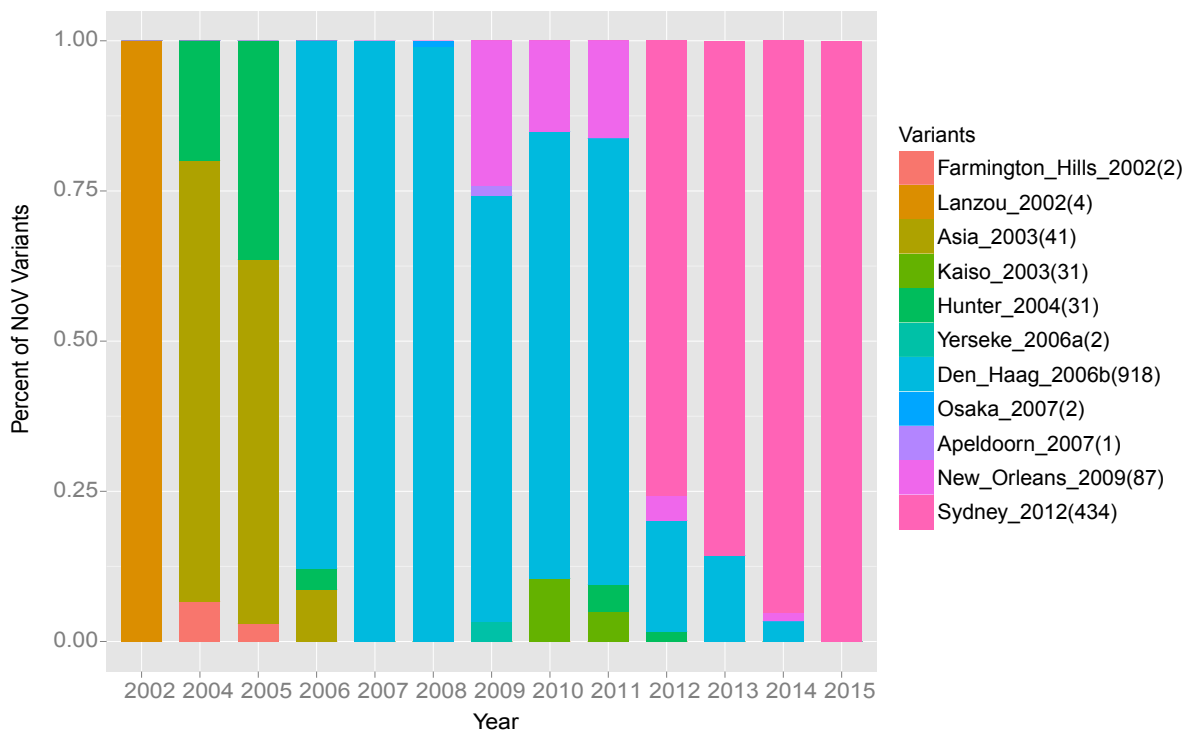

Supplement: Supplementary file 1 — GenBank accession numbers and Bayes factor test results of GI and GII NoVs sequences used in this study. (PDF 395 kb) [file 12920_2017_287_MOESM1_ESM.pdf]

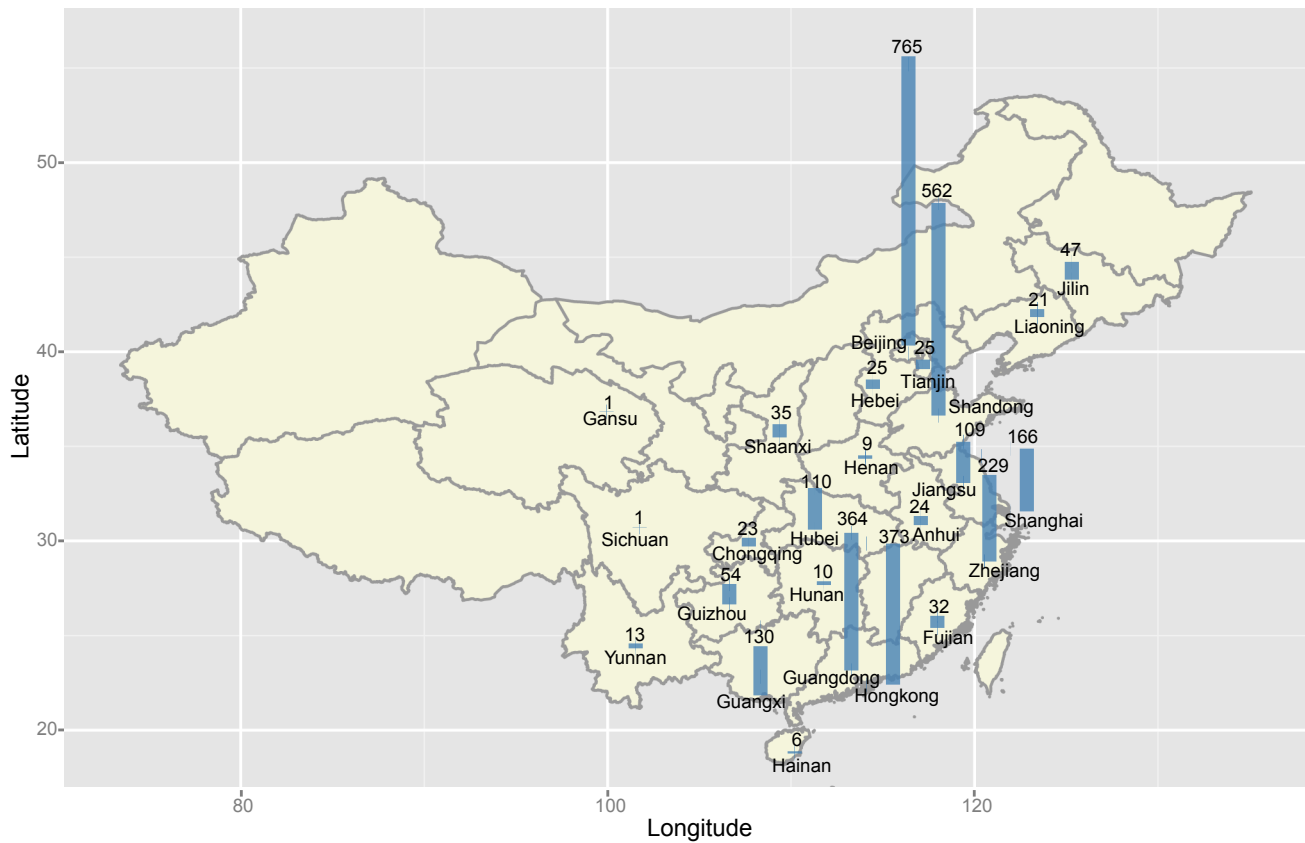

Supplement: Supplementary file 2 — KML file for NoV migration among locations of China over time as inferred from GI. Can be opened with Google Earth for visualization or any text editor or editing. (PDF 720 kb) [file 12920_2017_287_MOESM2_ESM.pdf]
